# Gastrointestinal neuroendocrine neoplasms (GI-NENs): hot topics in morphological, functional, and prognostic imaging

**DOI:** 10.1007/s11547-021-01408-x

**Published:** 2021-08-24

**Authors:** Ginevra Danti, Federica Flammia, Benedetta Matteuzzi, Diletta Cozzi, Valentina Berti, Giulia Grazzini, Silvia Pradella, Laura Recchia, Luca Brunese, Vittorio Miele

**Affiliations:** 1 Department of Radiology, Azienda Ospedaliero-Universitaria Careggi, Largo Brambilla 3, 50134 Florence, Italy; 2 Department of Nuclear Medicine, Azienda Ospedaliero-Universitaria Careggi, Largo Brambilla 3, 50134 Florence, Italy; 3 Department of Medicine and Health Sciences, University of Molise, 86100 Campobasso, Italy

**Keywords:** Neuroendocrine neoplasms, Gastrointestinal tract, Pathological correlation, Contrast-enhanced computed tomography, ^68^Ga-DOTA-peptides PET, TC, Prognostic imaging

## Abstract

Neuroendocrine neoplasms (NENs) are heterogeneous tumours with a common phenotype descended from the diffuse endocrine system. NENs are found nearly anywhere in the body but the most frequent location is the gastrointestinal tract. Gastrointestinal neuroendocrine neoplasms (GI-NENs) are rather uncommon, representing around 2% of all gastrointestinal tumours and 20–30% of all primary neoplasms of the small bowel. GI-NENs have various clinical manifestations due to the different substances they can produce; some of these tumours appear to be associated with familial syndromes, such as multiple endocrine neoplasm and neurofibromatosis type 1. The current WHO classification (2019) divides NENs into three major categories: well-differentiated NENs, poorly differentiated NENs, and mixed neuroendocrine-non-neuroendocrine neoplasms. The diagnosis, localization, and staging of GI-NENs include morphology and functional imaging, above all contrast-enhanced computed tomography (CECT), and in the field of nuclear medicine imaging, a key role is played by ^68^Ga-labelled-somatostatin analogues (^68^Ga-DOTA-peptides) positron emission tomography/computed tomography (PET/TC). In this review of recent literature, we described the objectives of morphological/functional imaging and potential future possibilities of prognostic imaging in the assessment of GI-NENs.

## Introduction

Neuroendocrine neoplasms (NENs) are epithelial tumours that originate from the diffuse endocrine system cells. They share the expression of neuroendocrine markers (such as chromogranin A and synaptophysin) and can secrete multiple different aminoacids and polypeptides, which may result in several clinical syndromes [1]. The neuroendocrine system is distributed over several organs, so NENs have many different denominations [2]: medullary carcinoma in thyroid, carcinoid, large-cell neuroendocrine carcinoma and small-cell carcinoma in lung and thymus, paraganglioma in extra-surrenal paraganglia, pheochromocytoma in adrenals and Merkel cell carcinoma in the skin.

NENs are most frequent in gastrointestinal tract (about 70% of cases), followed by lung (around 25% of cases) and pancreas [3].

Gastrointestinal neuroendocrine neoplasms (GI-NENs) are rather uncommon, representing around 2% of all gastrointestinal tumours and 20–30% of all primary neoplasms of the small bowel [4, 5]; they derive from enterocromaffin cells and are predominantly situated in the distal ileum [5, 6].

Clinically, we can classify NENs into non-functioning and functioning tumours. The functioning tumours secrete substances that cause appreciable clinical symptoms, whereas the non-functioning neoplasms do not secrete any substance or the substance produced is inactive. NENs can be sporadic, be the only manifestation of a disease, or be part of a multiple endocrine neoplasm (MEN), together with other endocrine tumours [2]. In addition to MEN, GI-NENs can associate with other genetic syndromes such as Von Hippel–Lindau and Neurofibromatosis type 1 [3]. At the time of diagnosis, only 15% of patients have the typical carcinoid syndrome, characterized by facial flushing, diarrhoea, or bronchospasm due to serotonin hypersecretion; otherwise, patients who present non-specific symptoms, such as weight loss, bleeding, and abdominal pain, are asymptomatic [7–9]. Carcinoid syndrome can facilitate NENs’s detection, despite many patients have advanced disease at diagnosis with distant metastases, mainly to the regional lymph nodes, liver, and bone [10–12]. Just over half of GI-NENs are diagnosed in the emergency setting with symptoms of gastrointestinal obstruction or bleeding [4].

According to the current World Health Organization (WHO) 2019 classification, based on the histopathological findings (Ki-67 index and mitotic rate), GI-NENs can be divided into three categories: well-differentiated NENs, poorly differentiated NENs, and mixed neuroendocrine-non-neuroendocrine neoplasms (MiNEN). Well-differentiated NENs are divided into grade 1 (Ki-67 index < 3, mitotic rate < 2), grade 2 (Ki-67 index 3–20, mitotic rate 2–20), and grade 3 (Ki-67 index > 20, mitotic rate > 20). Poorly differentiated NENs (Ki-67 index > 20% and mitotic index > 20) have a neuroendocrine component of more than 30% of the tumour. The last group consists of mixed neuroendocrine-non-neuroendocrine neoplasms (MiNEN) and has instead a non-neuroendocrine component of more than 30%; these may also be well differentiated or poorly differentiated [13].

Diagnostic imaging plays an important role in diagnosis, staging, and follow-up of GI-NENs [4, 14]. Several imaging techniques are used for this objective, in particular in the field of morphology imaging, we have abdominal ultrasound (US), contrast-enhanced computed tomography (CECT), and magnetic resonance imaging (MRI); for functional imaging, we rely on somatostatin receptor scintigraphy with ^111^Indium-pentetreotide (^111^In-Octreoscan),^68^Ga-labelled-somatostatin analogues (^68^Ga-DOTA-peptides) positron emission tomography/computed tomography (PET/TC), and ^18^FFluorodeoxyglucose-positron emission tomography (^18^F-FDG-PET) [11, 14].

In this review of recent literature, we aimed to evaluate the principal imaging features of GI-NENs to stratify patients into more defined clinical categories and to provide insights for precision medicine and appropriate treatments.

## Morphological imaging

Radiologic imaging plays a key role in the diagnosis, staging, and follow-up of GI-NENs [4, 14, 15].

The main techniques involved in abdominal radiology are ultrasound (US), contrast-enhanced computed tomography (CECT), and magnetic resonance imaging (MRI); this type of imaging is used to determine the size and location of primary tumour, as well as for staging and for evaluation of potential treatment choices.

**Fig. 1:**
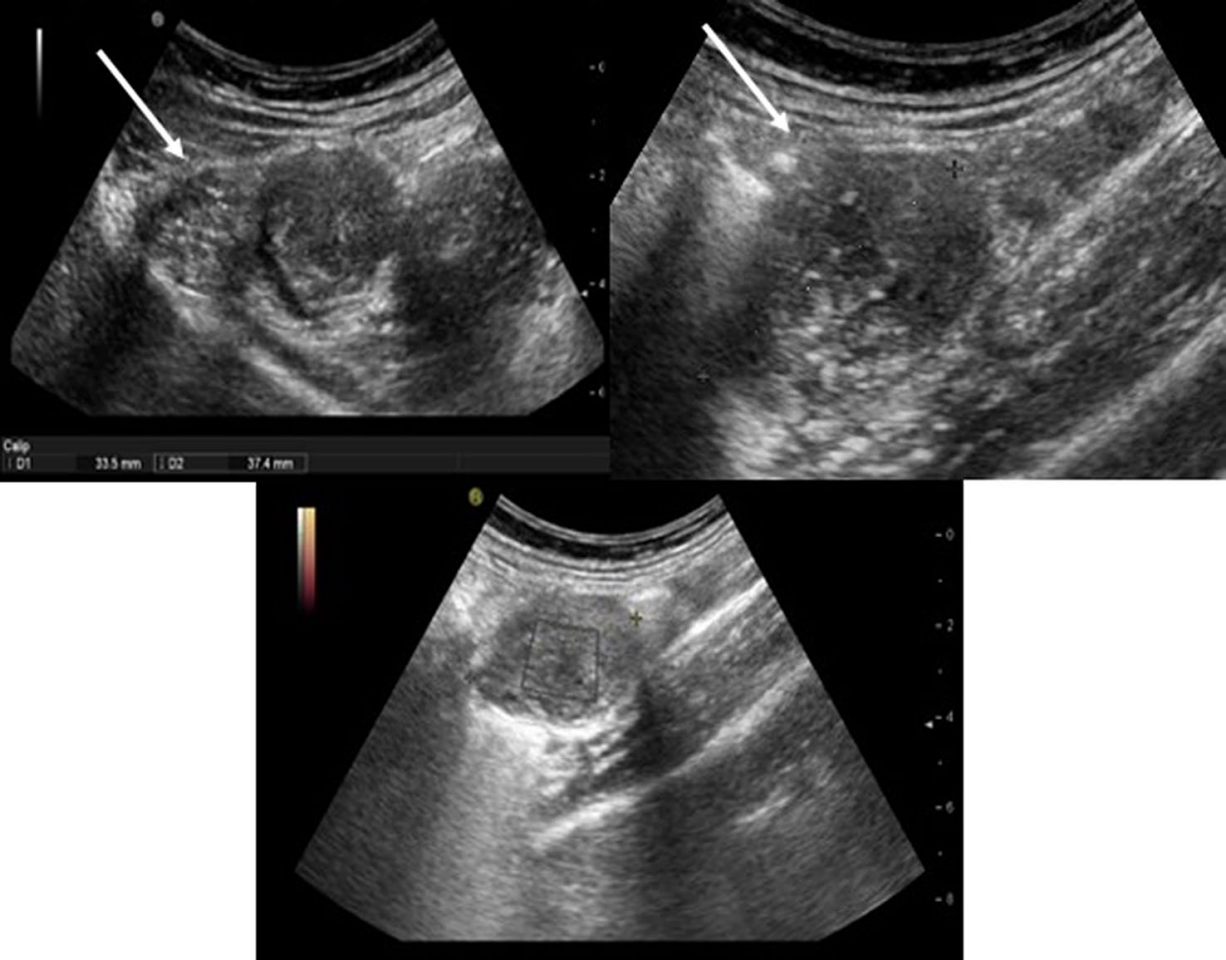
Gastrointestinal neuroendocrine neoplasms (G1). Transabdominal ultrasonography demonstrates a mass involving the appendix as a hypoechogenic lesion with a hyperechogenic border (white arrow)

Abdominal ultrasound is a radiation-free imaging examination [16, 17], but has a restricted role in the evaluation of gastrointestinal disease [18, 19]. Depending on location, sensitivity of abdominal US in revealing GI-NENs has variable percentage values (around 12–28%) [20]. Walczyk et al. reported how GI-NEN appears as a mass arising from the walls of gastrointestinal tract, and it may occur as hypoechoic lesions with a hyperechoic rim or as hyperechoic lesions (Fig. 1) [20, 21]. However, US examination is limited by operator dependence, it is not a panoramic method and visualization is confined to the field of investigation [20].

**Fig. 2:**
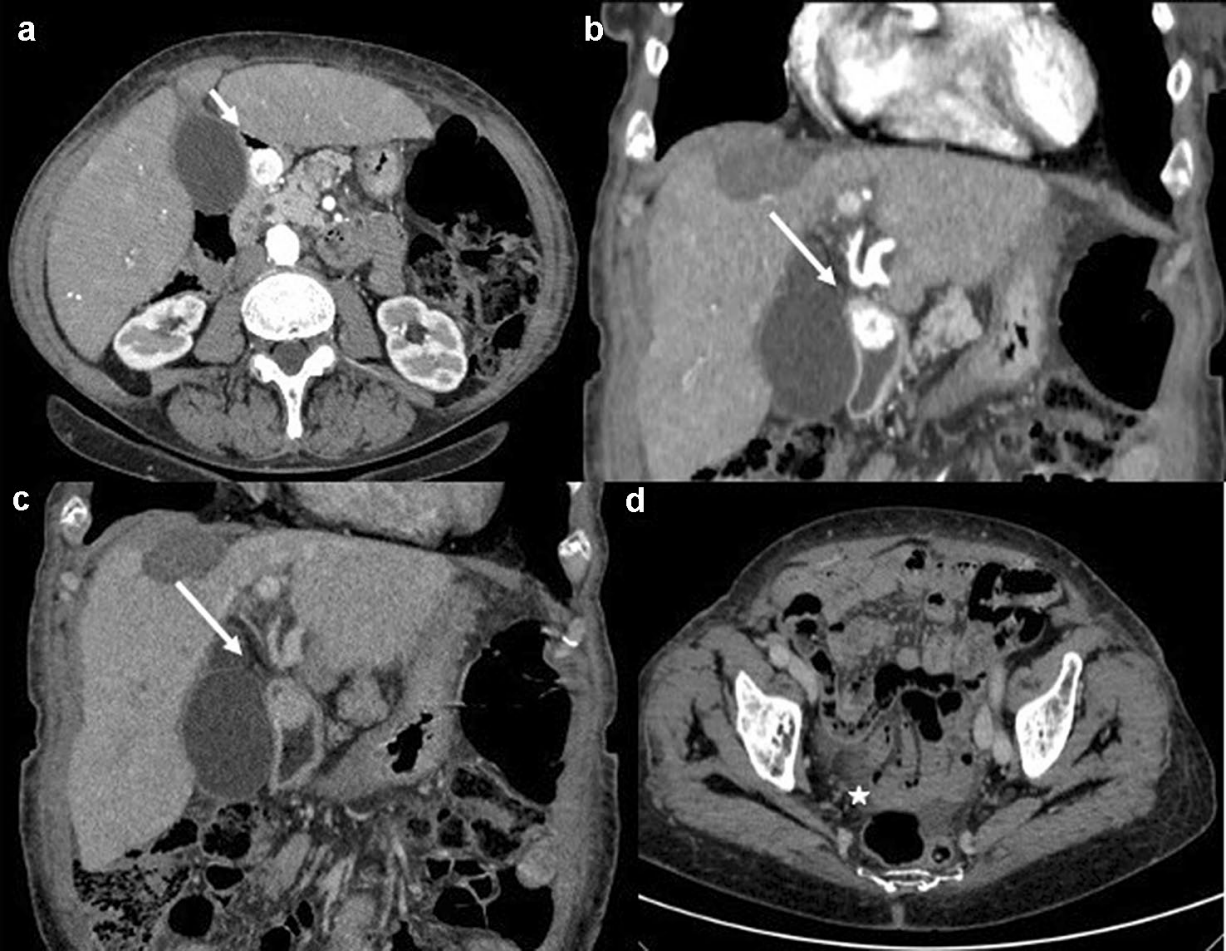
Gastric neuroendocrine neoplasm (G1). Axial and coronal contrast-enhanced CECT images in the arterial and portal venous phases (**a**, **b**, **c**) demonstrate an hypervascular intraluminal nodular mass of the gastric antrum (white arrow). In the inferior abdomen (**d**), ascites is appreciated (white star)

**Fig. 3:**
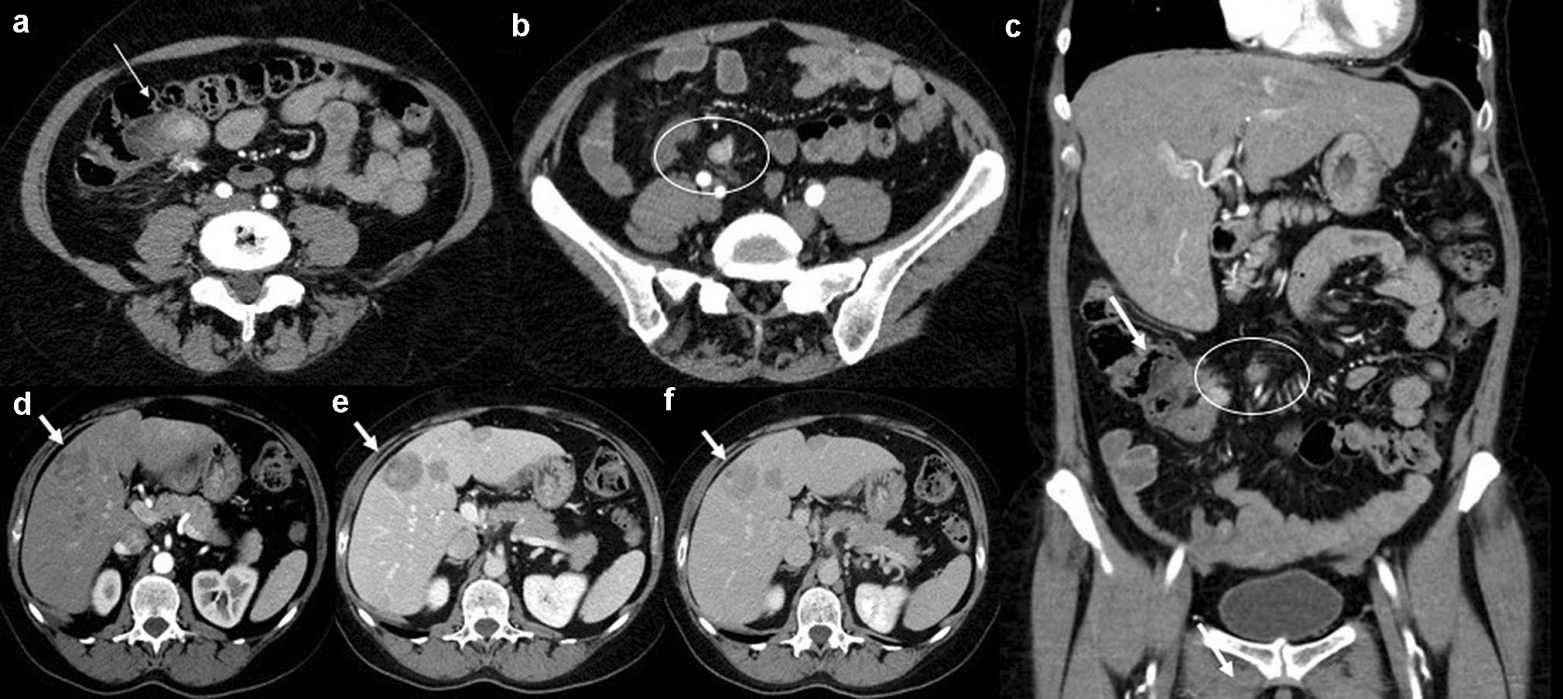
Gastrointestinal neuroendocrine carcinoma (NEC) in the terminal ileum. Axial (**a**, **b**) and coronal (**c**) contrast-enhanced CECT images in the arterial phase demonstrate a well-circumscribed enhancing mass (white arrows) of the terminal ileum involving ileocecal valve; in the mesenteric fat near the primary tumour, there is a mesenteric mass (**b**, **c**. white circle) with desmpplastic reaction. Arterial phase (**d**), portal venous phase (**e**) and equilibrium phase (**f**) contrast-enhanced MDCT show multiple hypervascular liver metastases (with arrows) with central necrosis

**Fig. 4:**
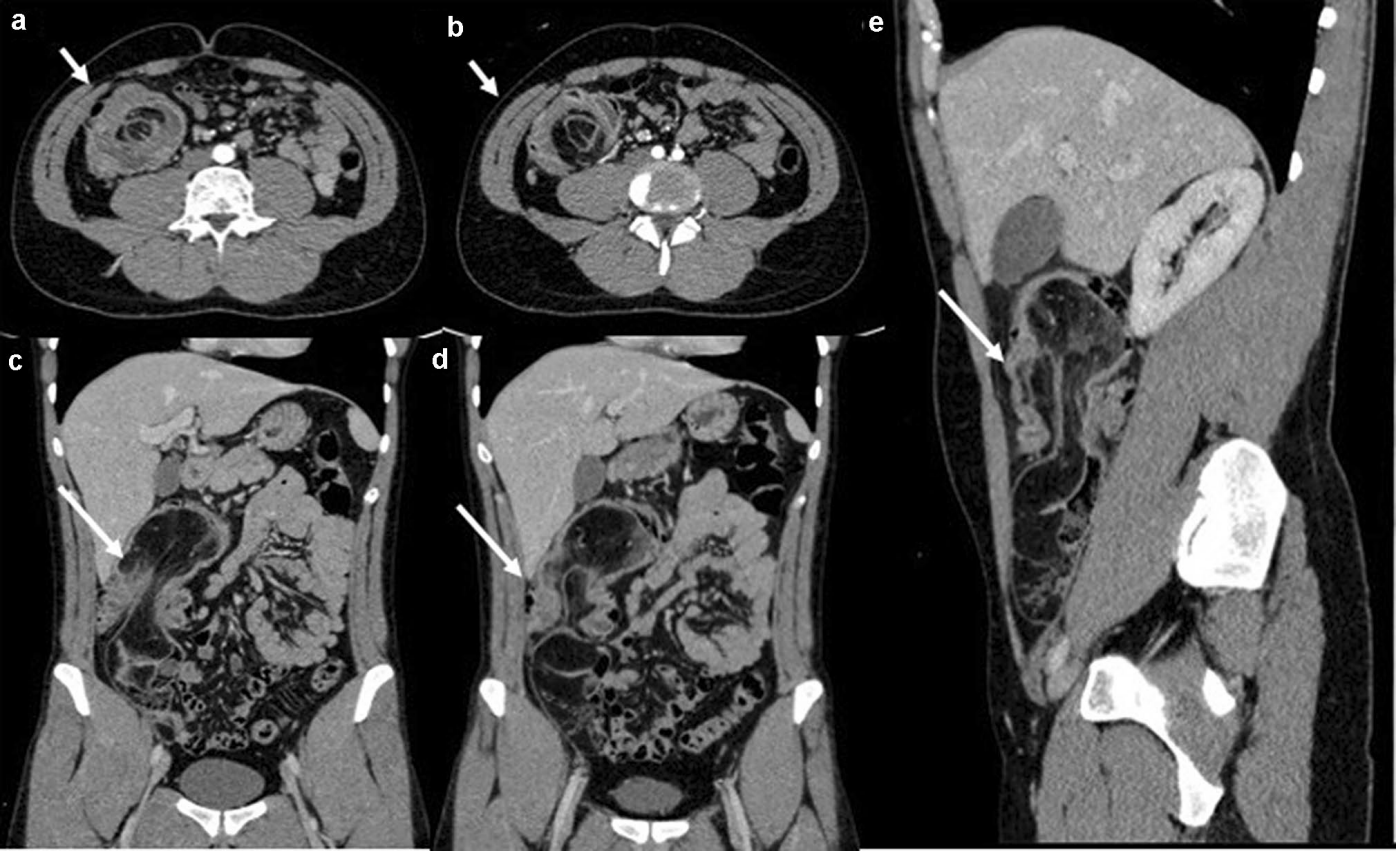
Gastrointestinal neuroendocrine neoplasm (G3) in the cecum. Axial (**a**, **b**), coronal (**c**, **d**) and sagittal (**e**) CECT image in the arterial (**a**, **b**) and portal venous phase (**c**, **d**, **e**) demonstrate an irregular bowel wall thickening of the cecum-ascendent colon, with intestinal intussusception (white arrows)

To date, the most widely used imaging modality for the detection of GI-NENs is undoubtedly CECT [4, 22–26], which allows diagnosis, staging, pre- and post-treatment evaluation and follow-up of these neoplasms (Fig. 2). A standard CECT protocol can be employed for both diagnosis and follow-up; patients can be scanned in the supine position with craniocaudal apnoea scans and should undergo non-contrast and contrast-enhanced CT scanning. Iodinated contrast medium can be injected into the antecubital vein at a flow rate of 3–4 mL/s using an automatic injector, immediately followed by a saline flush (40–50 mL). Contrast-enhanced biphasic images can be achieved during the arterial phase (30–35 s after injection onset) and the portal venous phase (70–75 s after injection onset) [27]. Enterographic computed tomography is a hybrid procedure that combines the techniques of intubation-infusion of small bowel imaging with those of conventional abdominal CT [28–30]. CT enterography has been shown to be useful in depicting small bowel parietal abnormalities and to be superior to conventional CT in this field; it has already been described by Kamaoui et al. that the sensitivity of CT enteroclysis in identifying small bowel NENs is 100% with a specificity of 96% [31]. At the time of diagnosis, approximately, half of patients with GI-NENs manifest advanced disease with metastases, most frequently localized to loco-regional lymph node stations, liver parenchyma, or bone [12–15] (Figs. 3, 4). The imaging appearance of NENs is widely variable, and it depends on localization, size, and margins, relationship to the bowel wall and the presence of intralesional alterations (haemorrhage, calcifications, necrosis, cystic degeneration, and ulceration) that modify its densitometric homogeneity and contrastographic pattern [4, 11]. After contrast medium injection, these gastrointestinal neoplasms may appear as a hypervascular nodular swelling originating from the intestinal wall or as a localized thickening of the wall [11] (Fig. 4). The boundary between the tumour and the adjacent tissue may be well or poorly defined; indeed, these lesions may have smooth, regular margins or jagged and irregular edges [4, 11].

**Fig. 5:**
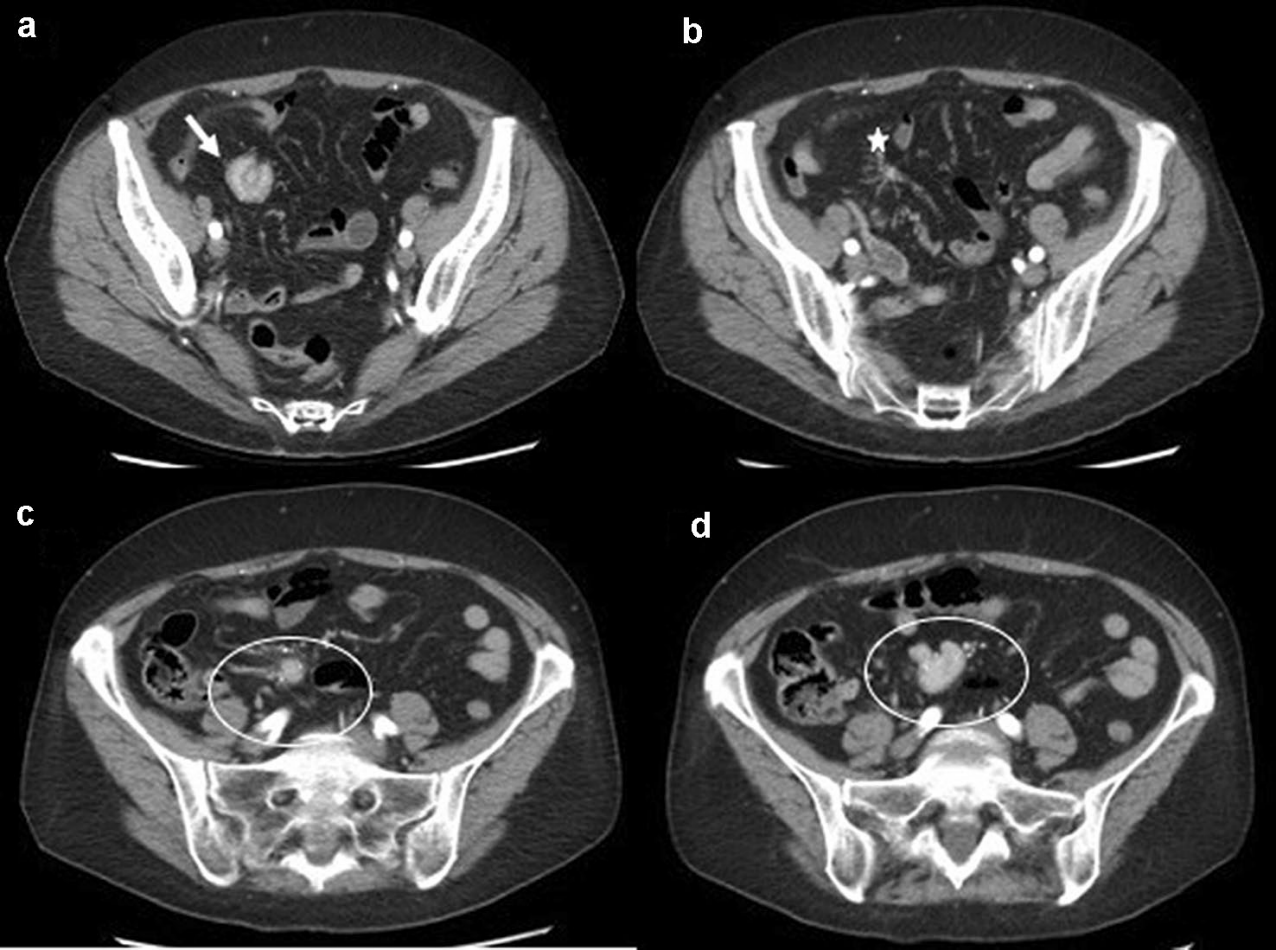
Gastrointestinal neuroendocrine neoplasm (G3) in the terminal ileum. Axial contrast-enhanced CT image in the arterial phase (**a**) demonstrates a bowel wall thickening (white line) of the terminal ileum and (**b**) a stellate soft tissue nodule (white star) in the mesentery. Under, there are many lymph nodes metastases (white circle) (**c**, **d**)

They are typically solid, but may have intralesional necrosis or cystic degeneration and, frequently, coarse or thin calcifications. Among the extra-intestinal manifestations of GI-NENs, the most typical is mesenteric metastasis associated with a desmoplastic reaction; this metastasis presents as a solid mass in the mesenteric fat adjacent to the primary neoplasm, associated with soft tissue rays, that radiate from the central mass to the small bowel. These extra-intestinal signs can cause complications such as kinking or angulation of bowel [4]. Other manifestations detectable at CECT are invasion of adjacent organs, omental/peritoneal involvement, metastases (lymphnode and liver), and ascites [12, 13] (Fig. 5). Many studies have demonstrated the correlation between CT features and pathological classification, revealing statistically significant differences between well-differentiated NENs and poorly differentiated NENs in terms of lesion size, growth, intra- or extra-intestinal involvement, zone of intralesional alterations, mesenteric fat infiltration and metastases; indeed, the finding on CT examination of lesions larger than 4 cm with necrotic or cystic areas in context, circumferential growth with transmural invasion, extra-intestinal involvement with mesenteric fat infiltration and lymphadenopathies, is more common in poorly differentiated NENs [4].

**Fig. 6:**
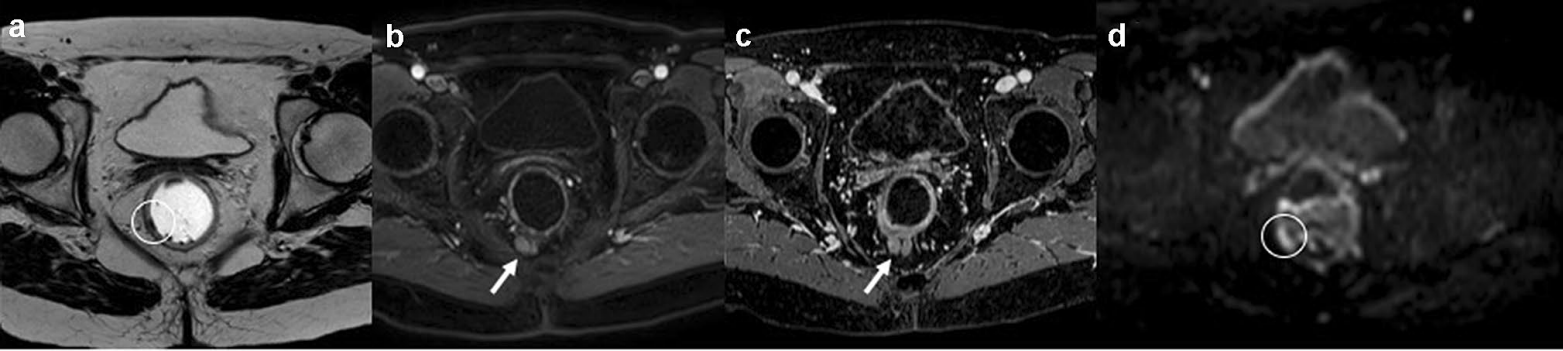
Gastrointestinal neuroendocrine carcinoma of rectum (NEC); MRI axial and coronal T2w imaging (**a**, **e**), T2w FAT-SAT, dynamic contrast-enhanced imaging (**c**) and DwI (**d**) demonstrate a thickening of right wall of rectum (white arrow and white circle)

Magnetic resonance imaging has an excellent soft tissue contrast [32–34] and, as abdominal US, it is a radiation-free imaging examination. The standard protocol for the evaluation of GI-NENs provides T1 and T2-weighted sequences, diffusion-weighted imaging (DWI), and dynamic contrast-enhanced imaging after administration of contrast medium [35–37]. Usually, GI-NENs appear hypointense on T1 and hyperintense on T2-weighted images, demonstrate a contrast enhancement pattern similar to that of CECT [8], and show diffusion restriction on DWI [38] (Fig. 6). Nevertheless, MRI is less used for the evaluation of patients with suspected GI-NENs because it is a time-consuming analysis and for its susceptibility to movement artefacts.

## Functional imaging of GI-NENs

Nuclear medicine study aids morphological imaging in the assessment of GI-NENs and that is allowed since common feature of most GI-NENs is the expression of somatostatin receptors (SSTRs) [39, 40]. This characteristic is exploited by nuclear medicine imaging to detect sites of disease, using radio-labelled SST analogues (SSA). For functional imaging of GI-NENs, we use somatostatin receptor scintigraphy with ^111^Indium-pentetreotide (^111^In-Octreoscan), ^68^Ga-labelled-somatostatin analogues (^**68**^Ga-DOTA-peptides) positron emission tomography/computed tomography (PET/TC) and ^18^FFluorodeoxyglucose-positron emission tomography (^18^F-FDG-PET).

^**111**^Indium-pentetreotide (Octreoscan®) uses a radio-labelled somatostatin analogues that detects neuroendocrine neoplasms by binding to characteristic somatostatin receptors displayed on the surface of GI-NEN cells [41–43]. Following administration of ^**111**^Indium-pentetreotide, whole-body scintigraphy is performed to diagnose, localize, and stage GI-NENs, but at the same time, it is important to remember that a significant subgroup of NENs is somatostatin receptor-negative. For GI-NENs, these scans have associated positive predictive values of 84.6% and negative predictive values of 50% [44, 45].

Krausz et al. in their study reported how Octreoscan® accuracy in diagnosis of liver metastases was 82%, this percentage is linked to several factors intrinsic to the method such as the low spatial resolution (usually around 1 cm) and long acquisition timings; moreover, reduced Somatostatin receptor scintigraphy detection of known lesions may be associated with a lower density or absence of receptors [46].

A fundamental and innovative role in the identification of GI-NENs has been the use of 68Ga-labelled somatostatin analogues (^68^Ga-DOTA-peptides) in positron emission tomography/computed tomography (PET/CT). Respect to Somatostatin receptor scintigraphy, ^68^Ga-DOTA-peptides have a higher sensitivity and spatial resolution and provide the possibility of image quantification [47–49].

**Fig. 7:**
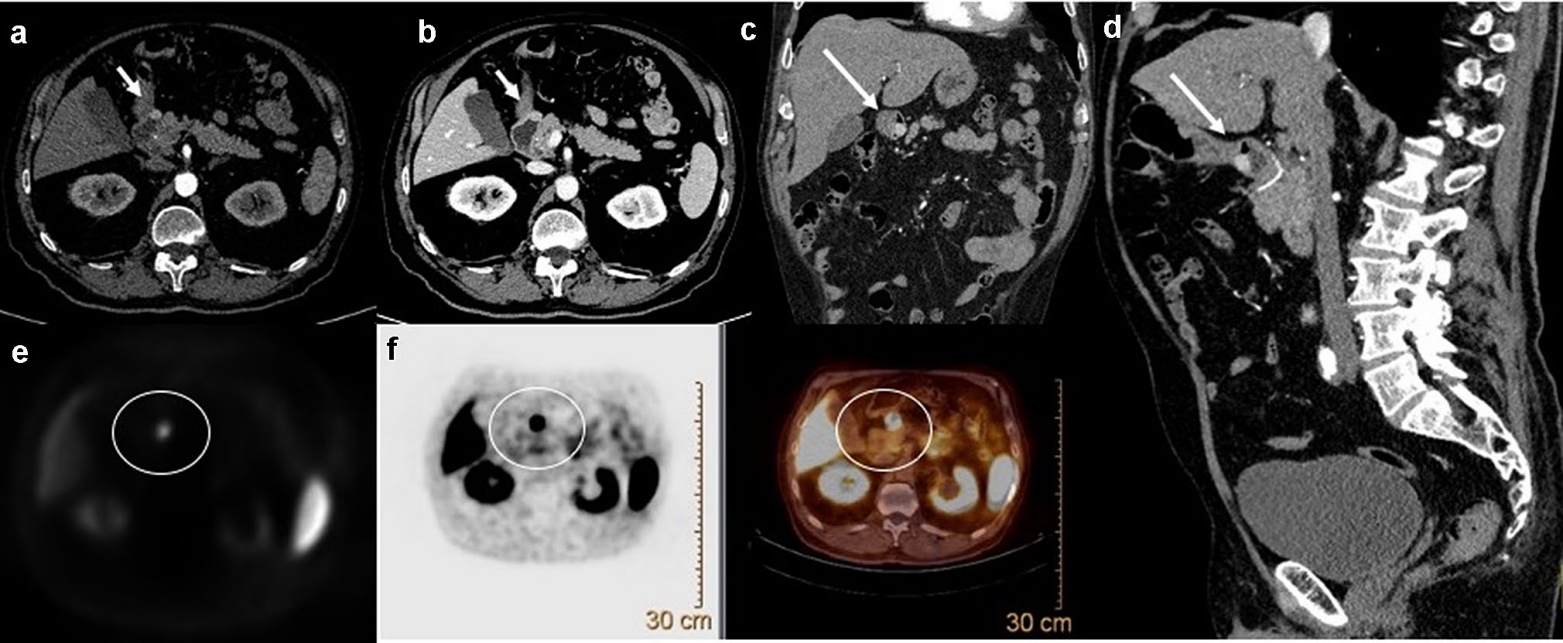
Gastrointestinal neuroendocrine neoplasms in the gastric antrum (G1). Axial (**a**, **b**) coronal (**c**) and sagittal (**d**) contrast-enhanced CECT images in the arterial (**a**, **c**, **d**) and venous portal phase (**b**) demonstrate a well-circumscribed ipervascular mass (white arrows) of the gastric antrum. At ^68^Ga-PET TC imaging (**e**, **f**) the lesion shows Ga uptake

**Fig. 8:**
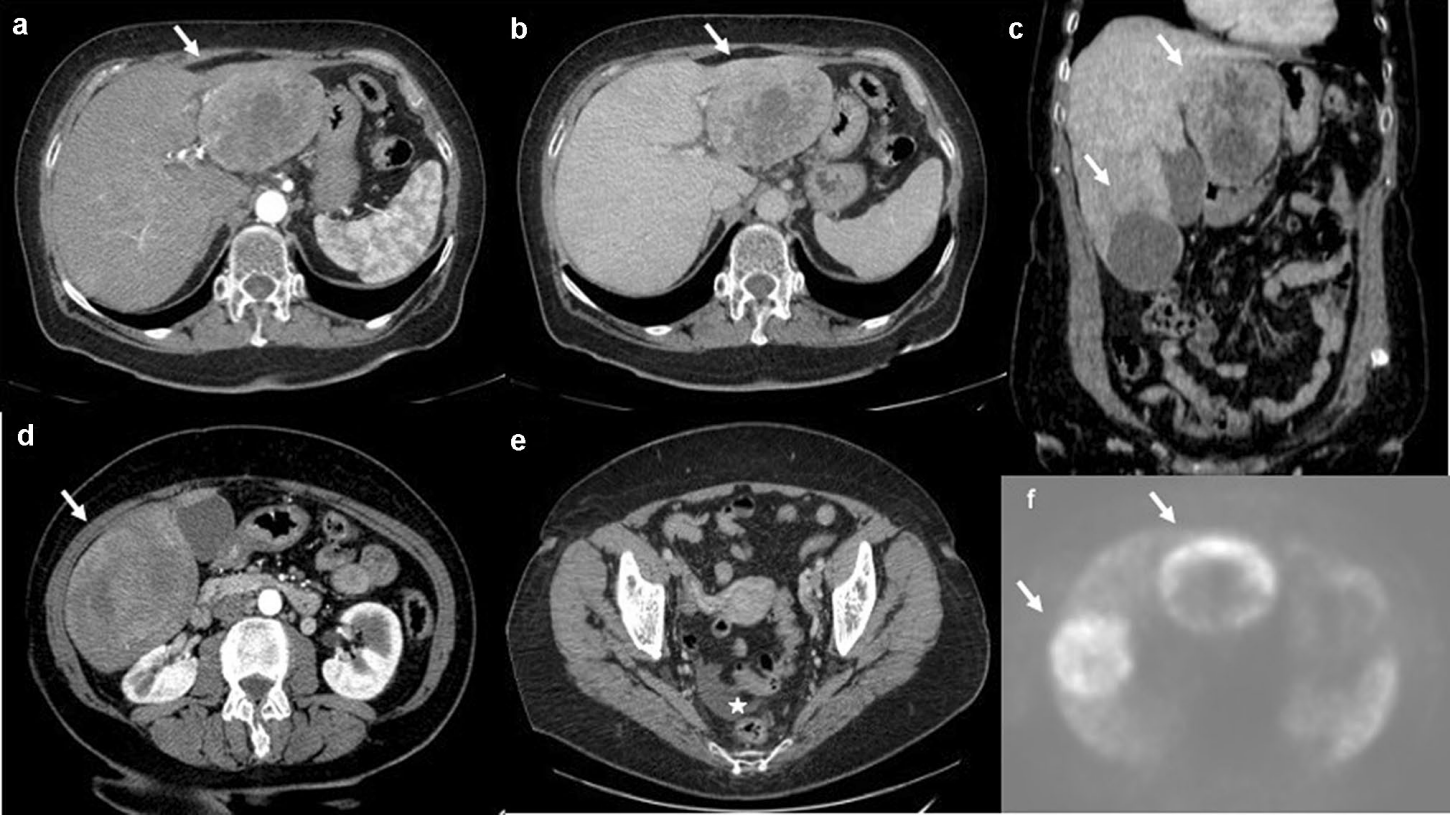
Arterial phase (**a**, **d**) and portal venous phase (**b**, **c**) contrast-enhanced CECT show multiple hypervascular liver metastases (withe arrows) of NEC, that become almost isodense on the portal venous phase except for central areas of necrosis. Ascites (**e**) is associated (withe star). At ^**68**^Ga-PET TC imaging (**f**) the liver lesions show Ga uptake

**Fig. 9:**
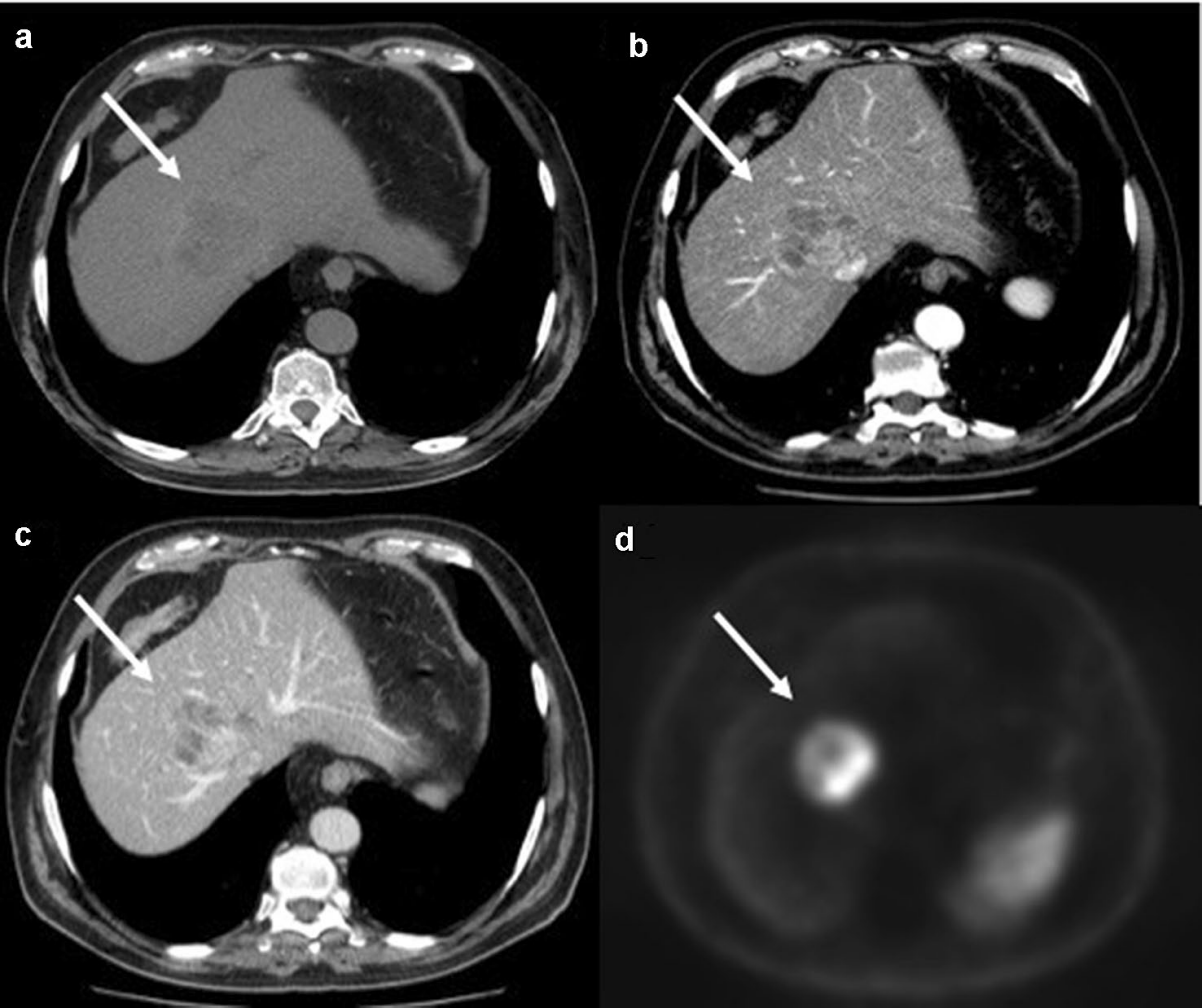
Axial pre-contrast image (**a**), arterial phase (**b**) and portal venous phase (**c**) contrast-enhanced CECT show hypervascular liver metastasis (white arrows) of NEC that becomes almost isodense on the portal venous phase except for central areas of necrosis. At ^**68**^Ga-PET TC imaging (**d**) the liver lesion shows Ga uptake

Recently, a lot of study have reported the diagnostic superiority of ^68^Ga-DOTA-peptides over other diagnostic investigation in the detection of GI-NENs [50, 51]. In particular, within of GI-NENs, ^**68**^Gallium-DOTA-peptides have been shown to have a better detection rate compared with CECT of primary tumour (89% vs 25%) and carcinomatosis (88% vs 75%), a higher detection rate than conventional Somatostatin receptor scintigraphy (95.1% vs 45.3%), and a lower radiation dose due to the shorter length of study (2 h); this method has also high sensitivity (93.5%) in the detection of liver metastasis from GI-NENs. [52, 53]. With the increased use of multiple radionuclide ligands, it is hoped that ^68^Ga-DOTA-peptides will have a crucial role in the future in assigning class risk stratification and data for personalized treatment in patients with GI-NENs (Figs. 7, 8, 9).

A new hybrid chelator (^68^Ga-DATA-peptides) has recently been introduced. This one has the advantage of radio-labelling at room temperature in lesser time compared to ^68^Ga-DOTA-peptides, with high radiochemical yield in a istant kit-type fashion. In particular, although ^68^Ga-DOTA-peptides are nowadays the current standard for imaging of GI-NENs, recent studies have shown that ^68^Ga-DATA-peptides’ elegant labelling profile is associated with diagnostic accuracy comparable to ^**68**^Gallium-DOTA-peptides [54, 55].

^18^FFluorodeoxyglucose-positron emission tomography allows to evaluate the metabolic activity of GI-NENs by studying the radio-labelled glucose metabolism. This method uses the increased glycolytic activity and increased expression of glucose transporters by tumour cells to identify these neoplasms compared to non-pathological tissue. [56]. A lot of study reported how ^18^F-FDG-PET has a limited role in the assessment of well-differentiated GI-NENs, but can be valuable for poorly differentiated GI-NENs [57, 58]. This is due to the fact that well-differentiated GI-NENs are more likely to express somatostatin receptors with high density, while poorly differentiated GI-NENs may display a lower density of somatostatin receptors but are more metabolically active, thus making them better visualized by ^18^F-FDG-PET. Additionally, Panagiotidis et al. in their study reported how the presence of increased glucose in GI-NENs highlights an increased propensity for invasion and metastasis, and how ^18^F-FDG-PET accordingly has higher sensitivity in delineating disease extend, mainly in aggressive and high-grade tumours [58].

## Prognostic imaging of GI-NENs

In the new era of precision medicine, radiomics is an emerging domain of research aiming to extract mineable high-dimensional data from diagnostic images (mainly from CECT, PET or MRI) [59]. The notion underlying the process is that both morphological and functional images contain qualitative and quantitative information, which may reflect the underlying pathophysiology of a tissue. Radiomics’ analyses can be performed in tumour regions, metastatic lesions, as well as in normal tissues [60]. The search method uses the following steps: acquisition of CT/RM/PET images of diagnostic quality, segmentation of neoplasm with definition of ROI of interest, extraction of radiomics features from the whole segmented volume or alternatively of some ROIs by appropriate software. The extracted features are generally divided into four categories: descriptive like shape, size, location; first-order statistics such as intensity histograms that relate pixel intensity (x-axis) to the number of pixels (y-axis) with analysis of mean, median, standard deviation, quartiles, kurtosis, skewness; second-order statistics concerns the analysis of the textures obtained, also known as “Haralick descriptors”; higher-order statistics represent repetitive and non-repetitive patterns extracted by Kernel transforms. Finally, statistical analysis and “data mining” are carried out to obtain diagnostic, predictive, and prognostic models by machine learning [61]. Several recent studies have begun to test the additional possibilities of quantitative imaging in many areas of abdominal oncology [62–69], but to the best of our knowledge, there are no known articles on this subject for GI-NENs. It would be interesting in the near future to extend quantitative imaging studies to this type of neoplasms, since the use of radiomics and texture analysis could be promising also in GI-NENs for more accurate diagnosis and differentiation, tumour risk stratification, management and assessment of treatment response.

## Conclusions

In conclusion, morphological imaging plays a fundamental role in diagnosis, staging, and post-treatment follow-up of GI-NENs; functional imaging allows not only to detect the neoplasm but also may be useful in predicting differentiation grade of GI-NENs. One of the possible further developments for GI-NENs could be the evaluation of quantitative imaging with texture analysis, radiomic and radiogenomic features that may be useful in the prognostic assessment of these tumours. In fact, in GI-NENs, the main clinical question concerns the need of the patient risk stratification. Therefore, together with the overall clinical profiles (morphological and functional imaging, symptoms and individual risk for evolving disease), prognostic imaging could potentially be a decisional factor to stratify patients into more defined clinical categories for precision medicine and appropriate treatment.
